# Assessing the need for coronary angiography in high-risk non-ST-elevation acute coronary syndrome patients using artificial intelligence and computed tomography

**DOI:** 10.1007/s10554-024-03283-9

**Published:** 2024-11-08

**Authors:** Aurelien Cagnina, Adil Salihu, David Meier, Wongsakorn Luangphiphat, Benjamin Faltin, Ioannis Skalidis, Aurelia Zimmerli, David Rotzinger, Salah Dine Qanadli, Olivier Muller, Emmanuel Abbe, Stephane Fournier

**Affiliations:** 1 Cardiology Department, University Hospital of Lausanne and University of Lausanne, Rue de Bugnon 46, Lausanne, 1011 Switzerland; 2 Institute of Mathematics, School of Computer and Communication Sciences, EPFL, Lausanne, Switzerland; 3 Radiology Department, University Hospital of Lausanne, Lausanne, Switzerland

**Keywords:** Artificial intelligence, ChatGPT, machine learning, large language models

## Abstract

**Purpose:**

This study aimed to evaluate the efficacy of the Chat Generative Pre-trained Transformer (ChatGPT) in guiding the need for invasive coronary angiography (ICA) in high-risk non-ST-elevation (NSTE) acute coronary syndrome (ACS) patients based on both standard clinical data and coronary computed tomography angiography (CCTA) findings.

**Methods:**

This investigation is a sub-study of a larger prospective multicentric double blinded project where high-risk NSTE-ACS patients underwent CCTA prior to ICA to compare coronary lesion by both modalities. ChatGPT analyzed clinical vignettes containing patient data, electrocardiograms, troponin levels, and CCTA results to determine the necessity of ICA. The AI’s recommendations were then compared to actual ICA findings to assess its decision-making accuracy.

**Results:**

In total, 86 patients (age: 62 ± 13 years old, female 27%) were included. ChatGPT recommended against ICA for 19 patients, 16 of whom indeed had no significant findings. For 67 patients, ChatGPT advised proceeding with ICA, and a significant lesion was confirmed in 58 of them. Consequently, ChatGPT’s overall accuracy stood at 86%, with a sensitivity of 95% (95% confidence interval (CI) 0.76–0.92) and a specificity of 64% (95% CI 0.62–0.94). The model’s negative predictive value was 84% (95% CI 0.44–0.79), and its positive predictive value was 87% 95% CI 0.86–0.97).

**Conclusion:**

Preliminary evidence suggests that ChatGPT can effectively assist in making ICA decisions for high-risk NSTE-ACS patients, potentially reducing unnecessary procedures. However, the study underscores the importance of data accuracy and calls for larger, more diverse investigations to refine artificial intelligence’s role in clinical decision-making.

## Central illustration

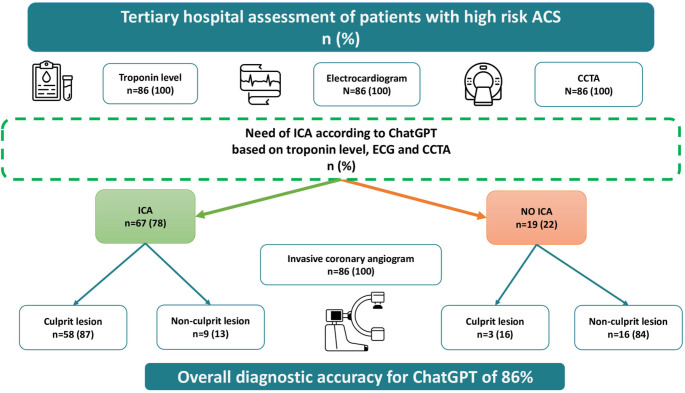

CCTA : coronary computed tomography angiography; ECG : electrocardiogram; ICA : invasive coronary angiogram; NSTE-ACS : non-ST-elevation acute coronary syndrome.

## Introduction

Managing high-risk acute coronary syndrome (ACS) patients often presents a significant challenge, as clinical assessment, electrocardiogram (ECG), and blood tests such as troponins can be insufficient for a definitive and final diagnosis. As a result, up to 30% of the patients undergo unnecessary invasive coronary angiography (ICA), exposing them to potentially avoidable procedural complications while increasing costs and resource use [1]. 

Coronary CT angiography (CCTA) is emerging as a potential non-invasive tool that can help refine selection of patients requiring ICA but its role remains unvalidated in this context. In addition, since none of the currently available tools are perfect and since results can sometimes be contradictory, clinicians are often faced with a dilemma when choosing between all the results of the diagnostic armamentarium in order to decide whether a patient requires ICA.

Artificial intelligence (AI)-based language models, such as the OpenAI Chat Generative Pre-trained Transformer (ChatGPT), have the potential to integrate and analyze vast amounts of data and provide valuable insights for clinical decision-making as assessed by two previous studies [2, 3]. The present study aimed to evaluate the potential of ChatGPT in guiding decisions on ICA recommendation in high-risk non-ST-elevation (NSTE) ACS patients who have undergone a CCTA before ICA.

## Methods

This is a sub-study of a larger prospective double blinded multicentric study that involved patients presenting with symptoms of ischemia and diagnosed with high-risk NSTE-ACS. This project tested the ability of CCTA in evaluating angiographic FFR against invasive FFR [4] This former study was approved in May 2019 by the Medical Ethical Committee of the Canton of Vaud (CER-VD) and is also registered on ClinicalTrials.gov (NCT04052763). Informed consent was taken in all patients included between 2020 and 2022 from this larger study. All patients from our institution were included in this present analysis without any exceptions and had an indication for invasive coronary angiogram according to current guidelines [1, 5]. High risk NSTE-ACS were defined by a rise and fall in troponin levels and at least one of the following: symptoms of ischemia or ST/T wave changes. Patients with STEMI were excluded or with very high-risk criteria according to current guidelines. All patients underwent a clinical history, physical examination, ECG, troponin level measurement, CCTA, and ICA. In every 30–90% stenosis, invasive hemodynamic evaluation was performed with measurement of Fractional Flow Reserve. Further details can be found directly in the description of the study [4]. 

A programmed script automatically generated patient vignettes from a standardized spreadsheet of anonymized clinical data, including symptoms based on a predefined checklist of common and frequently encountered terms, ECG description, troponin values, and CCTA results, that were submitted to the ChatGPT AI model. The standardised text that was used for each patient and that was submitted to ChatGPT was as follows “ A [XX]-year-old [male/female] patient with a known history of [risk factor] presents to the emergency department with [symptoms]. Laboratory results indicate an elevation of troponin to [XX] ng/L. The ECG reveals [XX], and the coronary computed tomography angiography demonstrates [XX]. Provide a recommendation on whether to proceed with invasive coronary angiography based on the available data.”

ChatGPT was asked to provide a recommendation on whether to proceed with ICA or not, based on the available data. Recommendations of the AI model were then compared with the actual angiography findings to assess the model’s performance in guiding clinical decisions for high-risk NSTE-ACS patients. Each vignette was written in a separate conversation to avoid bias and prevent influencing ChatGPT’s responses. The version of GPT-4 from March to April 2023 was employed.

### CCTA acquisition information

Image acquisition was performed on a 256-row multidetector CT system (Revolution CT, GE Healthcare, Milwaukee, WI, USA), with patients lying supine, arms raised above the head, in a single breath hold. Axial single-heartbeat mode CCTA with prospective ECG-gating was performed. Detailed CCTA parameters were as follows: tube potential, 80–120 kVp based on patient weight; tube load, 400–800 mA determined automatically based on a noise index of 20; gantry revolution time, 0.28 s; automatic exposure control (angular and longitudinal), no; beam collimation geometry, 256 × 0.625 mm. Reconstruction parameters were the following: field-of-view, 220–250 mm; matrix size, 512 × 512 pixels; slice thickness, 0.625 mm; slice incrementation, 0.625 mm; reconstruction kernel, standard; reconstruction algorithm, deep learning image reconstruction high. Images transferred to the manufacturer’s workstation (AW server, version 3.2) and reviewed offline by two cardiovascular radiologists with 10 and > 20 years of experience, respectively. The readers performed multiplanar and curvilinear reconstructions to quantify stenoses, determine high-risk plaque, as well as first-pass static rest myocardial perfusion as described by Pursani A et al. [6] Stenosis quantification was performed semi-automatically using lumen segmentation and diameter stenosis measurement, and reported according to the CAD-RADS 2.0 scheme.

### Statistical analysis

Data were summarized using descriptive statistics. For continuous variables with a normal distribution, the mean and standard deviation (mean ± SD) were reported. For continuous variables that were not normally distributed, the median and interquartile range (IQR) were used. Categorical variables were presented as frequencies and percentages. This study was not designed to perform advanced between-group comparisons. All analyses were conducted using SPSS Statistics (IBM).

## Results

A total of 86 patients were included. The mean age was 62 ± 13 years. The median troponin peak value was 35 [46, 370] ng/l, and 52 patients had ECG findings compatible with ischemia. Baseline characteristics of the study population are displayed in Table 1 with characteristics according to ICA recommendation by ChatGPT and culprit lesion in Tables 2 and 3. Information reported in Tables 1 and 4 were included in a prompt submitted to ChatGPT. CCTA revealed < 30% diameter stenosis (DS) in 15 patients, 30–49% DS in 10 patients and > 50% DS in 61 patients.

**Table 1:** Baseline characteristics of patients

	Overall *N* = 86
**Clinical data**	
Age, m ± SD (in years)	62 ± 13
Female	23 (27)
Type of symptomatology at presentation	
Chest pain n (%)	84 (98)
Shortness of Breath n (%)	38 (44)
Lightheadedness n (%)	11 (13)
Positive family history n (%)	21 (24)
Hypercholesterolemia n (%)	52 (60)
Hypertension n (%)	45 (52)
Diabetes Mellitus n (%)	12 (14)
BMI ≥ 30 kg/m^2^ n (%)	13 (15)
Prior myocardial infarction n (%)	1 (1)
Prior stroke n (%)	3 (3)
Peripheral artery disease n (%)	2 (2)
**Laboratory assessment**	
Troponin T hs level, median [IQR] (in ng/l)	35 [46, 370)
Creatinine kinase level, median [IQR] (in ng/l)	136 [96, 235]
**ECG assessment**	
Normal n (%)	34 (40)
ST depression n (%)	12 (14)
T wave inversion n (%)	29 (34)
T wave flattening n (%)	11 (13)
New RBBB n (%)	8 (9)

BMI : Body mass index ; RBBB : right bundle brunch block

**Table 2:** Baseline characteristics of patients according to ChatGPT’s decision and presence or absence of culprit lesion

	ICA recommendation by ChatGPT *N* = 67	No ICA recommadation by ChatGPT *N* = 19	Présence of culprit lesion *N* = 61	Absence of culprit lesion *N* = 25
**Clinical data**				
Female	14 (21)	9 (47)	12 (20)	11 (44)
Type of symptomatology at presentation				
Chest pain n (%)	65 (97)	19 (100)	59 (97)	25 (100)
Shortness of Breath n (%)	32 (48)	6 (32)	29 (48)	9 (36)
Lightheadedness n (%)	9 (14)	2 (11)	7 (11)	4 (16)
Positive family history n (%)	19 (28)	2 (11)	18 (30)	3 (12)
Hypercholesterolemia n (%)	43 (64)	9 (47)	42 (69)	10 (40)
Hypertension n (%)	37 (55)	8 (42)	36 (59)	9 (36)
Diabetes Mellitus n (%)	9 (13)	3 (16)	11 (18)	1 (4)
BMI ≥ 30 kg/m^2^ n (%)	11 (16)	2 (11)	10 (16)	3 (12)
Prior myocardial infarction n (%)	1 (1)	0 (0)	1 (2)	0 (0)
Prior stroke n (%)	3 (4)	0 (0)	3 (5)	0 (0)
Peripheral artery disease n (%)	2 (3)	0 (0)	2 (3)	0 (0)
**Laboratory assessment**				
Troponin T hs level, median [IQR] (in ng/l)	198 [16, 378]	66 [19, 135]	218 [16,423]	51 [19,96]
Creatinine kinase level, median [IQR] (in ng/l)	138 [45,195]	122 [49,287]	143 [45,255]	102 [47, 160]
**ECG assessment**				
Normal n (%)	21 (31)	13 (68)	20 (33)	14 (56)
ST depression n (%)	12 (18)	0 (0)	8 (13)	4 (16)
T wave inversion n (%)	27 (40)	2 (11)	24 (39)	5 (20)
T wave flattening n (%)	10 (15)	1 (5)	9 (15)	2 (8)
New RBBB n (%)	4 (6)	5 (55)	4 (7)	4 (16)

**Table 3:** Per patient level CCTA findings :

	Overall *N* = 86	ICA recommendation by ChatGPT *N* = 67	No ICA recommadation by ChatGPT *N* = 19
Number of lesions per patient	1 ± 0.9	1.3 ± 0.8	0.2
Angiographic CAD extent (> 50% stenosis)	61	60	1
No CAD	25	7	18
1 vessel	35 (35)	35 (58)	0
2 vessels	21 (24)	21 (30)	0
3 vessels	5 (6)	4	1
CAD-RADS score			
0	6	3	3
1	9	4	5
2	7	4	3
3	9	7	2
4	41	39	2
5	10	9	1

**Table 4:** Per lesion level CCTA findings

	Overall *N* = 86	ICA recommendation by ChatGPT *N* = 67	No ICA recommadation by ChatGPT *N* = 19
Number of lesion	69	68	1
Degree of stenosis			
Left main artery stenosis			
30–49%	2	2	0
50–69%	2	2	0
Left anterior descending artery stenosis			
Proximal (70 99%)	16	15	1
Mid (70–99%)	20	20	0
Distal (70–99%)	5	5	0
Circumflex artery stenosis			
Proximal (70 99%)	5	0	0
Mid (70–99%)	5	0	0
Distal (70–99%)	3	3	0
Right coronary artery stenosis			
Proximal (70 99%)	1	1	0
Mid (70–99%)	7	7	0
Distal (70–99%)	3	3	0

CAD : coronary artery disease, CCTA : coronary computed tomography angiography

Following ICA, 61 patients were deemed as having at least one “culprit” lesion that was treated, while 25 patients were declared as having non-obstructive coronary arteries (**central illustration)**. ChatGPT recommended against ICA in 19 patients. Among these 19 patients, 3 had actual stenoses. Two of these mistakes were induced by the CCTA, which failed to detect the stenoses, rather because of ChatGPT. The other mistake occurred in a 38-year-old woman with a pathological CCTA with description of a triple vessel disease (stenosis of 50–69% of distal RCA and mid Cx and 70–99% in 1st diagonal) but no traditional cardiovascular risk factors, for whom ChatGPT recommended against ICA.

Among the 67 patients for whom ChatGPT recommended ICA, 9 had no significant findings. Table 3 and 4 consolidate CCTA findings per patient and per lesion, respectively. The performance metrics of ChatGPT are presented in Table 5 and are delineated as follows: the positive and negative predictive values are 87% (95% Confidence Interval (CI) 0.86–0.97) and 84% (95% CI 0.44–0.79), respectively, while the sensitivity and specificity stand at 95% (95% CI 0.76–0.92) and 64% (95% CI 0.62–0.94), respectively. The comprehensive accuracy rate of ChatGPT is 86%.

**Table 5:** Performance of ChatGPT to assess the need for ICA

Sensitivity, *n* (%)	58/61 (95) (95% CI 0.76–0.92)
Specificity, n (%)	16/25 (64) (95% CI 0.62–0.94)
Positive predictive value n (%)	58/67 (87) (95% CI 0.86–0.97)
Negative predictive value, n (%)	16/19 (84) (95% CI 0.44–0.79

CI : confidence interval

## Discussion

To the best of our knowledge, this is the first study assessing the potential role of AI, specifically the ChatGPT model, to guide decisions on whether to perform ICA in high-risk NSTE-ACS patients who have undergone CCTA. The findings suggest that ChatGPT could offer valuable guidance in this challenging clinical scenario, where even with CCTA - that can reveal a wide range of findings, from normal arteries to occlusion, as well as many borderline lesions – the decision whether to perform ICA might not be straightforward.

A significant finding is that among the three patients with a culprit lesion where ChatGPT recommended against ICA, the model was misled by CCTA results that missed a lesion in two cases. In the third case, the lack of additional information on the type or physiology of the plaques may have influenced ChatGPT’s judgment. This underscores the dependence of AI accuracy on the quality of input data, highlighting the necessity of accurate and reliable data for training and testing AI models. Interestingly, in nine cases where ChatGPT recommended ICA, no coronary lesion or significant cardiovascular risk factors were present. This confirms the need for comprehensive input data to maximize the accuracy of AI recommendations. However, the ‘black box’ nature of AI decision-making, as mentioned in previous studies, remains a significant challenge, as the underlying logic of AI recommendations is often opaque [7]. Thus, the accuracy of the information fed to the AI model could potentially be enhanced through the use of other AI tools to interpret imaging to potentially improve the precision of the AI’s decision-making process.

Despite the lack of formal integration of AI into current clinical guidelines, its applications in cardiology are expanding rapidly. AI has shown promise in various imaging modalities and decision-making processes [8, 9]. Recently, we demonstrated that ChatGPT could potentially aid in decision-making process during heart-team meetings for patients with severe aortic stenosis through complex clinical scenarios involving only few variables [3]. Indeed, AI’s model treatment recommendation was compared with the decision of the Heart Team, and the model was in phase with the Heart Team in 77% of the situation. One aspect we highlight is the importance of the information provided to these Large Language Model (LLM) models, which could theoretically improve their accuracy. To enhance the specificity of this process, incorporating a refined CCTA analysis, as previously discussed, along with an analysis of plaque physiology and characterization, could be beneficial [10]. AI software could help identify and extract data from plaque burden, describe the presence of calcified or non-calcified lesions, and determine their degree of stenosis as reported in chronic coronary syndrome [11–13]. Subhi et al. found that predictive models based on CCTA could accurately identify future culprit lesions with a specificity of 89.3%, indicating the potential for AI to enhance predictive accuracy through detailed analysis [14]. Another recent study assessed the efficacy of an AI-based tool in assessing coronary stenosis by comparing measured lesion in CCTA against a benchmark standard (ICA), and found it to exhibit a high diagnostic accuracy [15]. Here, we can observe the potential of decision-making AI by taking into account the medical history, biological assessment, ECG, and the detailed results of the CTCA on a single prompt. This approach aims to deliver the highest quality of care to patients through the utilization of AI, while also accelerating diagnoses and treatment [9]. 

The use of CCTA in this acute phase has been demonstrated to be safe and helps to limit invasive examinations in emergency room [16]. The use of CCTA for patients with low to moderate risk of ACS has been incorporated into the latest guidelines due to its high negative predictive value [1, 17, 18]. In addition to its value as a “rule out” exam, CCTA is as effective as ICA in assessing long-term risk when performed in the acute phase [19]. The future lies also in the development of coronary physiology using CCTA, which has been applied in chronic coronary syndrome but not yet in ACS. A systematic review and meta-analysis showed a moderate agreement between FFR-computed tomography (CT) and ICA, with the highest agreement with invasive FFR values greater than 0.90. Our study will hopefully try to respond to that question in acute coronary syndrome [4]. 

Our study found reassuring results in the AI decision-making process, with an overall accuracy of 86%, which is excellent for a non-trained AI model, albeit with a specificity of 64%. The advent of current and future techniques will accelerate all processes involved in the management of these patients and will further improve the sensitivity and specificity of the decision-making process. However, we acknowledge certain limitations: the significance of CCTA-derived information in this context, where CCTA is not typically recommended, is unknown; the potential variability in ChatGPT’s responses has not been examined, though our aim was to simulate a real-life scenario where a physician might use ChatGPT as a consultative tool for decision-making. Moreover, this study was neither intended nor powered to compare diagnostic modalities directly; it was designed as a feasibility study to assess if AI could manage complex clinical situations and aid in decision-making.

Finally, in the preliminary phase of implementing AI in clinical settings, it is crucial not to blindly follow the advice of a LLM. LLMs do not possess real-time updates or a deep understanding, which could result in confident yet erroneous conclusions. Additionally, the complexity of AI decision-making and the opaque “black box” nature of its algorithms can obscure the logic behind its recommendations, posing significant concerns in critical fields such as healthcare. In the context of a prospective future study, a head-to-head comparison between physician and ChatGPT would allow for a better assessment of diagnostic accuracy. However, due to our study’s design, this could not be accomplished.

## Conclusion

This study offers preliminary evidence that a CCTA-based approach followed by analysis using AI models such as ChatGPT has the potential to facilitate clinical decision making regarding the need for ICA in high-risk NSTE-ACS patients. Larger, prospective studies with a more diverse patient population and more extensive input data, including other imaging modalities, clinical scores, and patient-reported outcomes, could help to refine and validate AI models in this setting. Additionally, ethical considerations should be evaluated when implementing AI in clinical practice.

## Data Availability

No datasets were generated or analysed during the current study.
